# CPAP caps are associated with restricted head growth and altered skull morphology in newborn infants

**DOI:** 10.3389/fped.2025.1514853

**Published:** 2025-05-19

**Authors:** Sebastian Jacob, Nancy Wetzel, Annett Bläser, Ulrich Herbert Thome, Rudolf Georg Ascherl

**Affiliations:** ^1^ Division of Neonatology, Department of Women’s and Children’s Health, University Hospital of Leipzig, Leipzig, Germany; ^2^ Department of Pediatrics, University Hospital of Halle-Wittenberg, Halle/Saale, Germany; ^3^ Department of Child and Adolescent Psychiatry, Psychosomatic Medicine and Psychotherapy, University Hospital of Jena, Jena, Germany

**Keywords:** CPAP (continuous positive airway pressure), neonatology, preterm (birth), head circumference, ear-to-ear (E2E), growth

## Abstract

**Background:**

Continuous positive airway pressure (CPAP) devices for preterm infants are commonly affixed using snug-fitting caps. Monitoring of head growth is standard practice in preterm infants, as stagnant head growth has been associated with impaired neurodevelopmental outcome. However, a stagnant head circumference may not mean stagnant head growth since vertical head distortion has been repeatedly observed. Previously established centiles for ear-to-ear distances and head volume indices allow the evaluation of three-dimensional head growth. We hypothesized that CPAP duration may be associated with restricted head circumference gain, altered skull morphology, and possibly neurodevelopment.

**Patients and methods:**

All 4590 infants treated with CPAP in the neonatal wards of Leipzig University Medical Center between 2009 and 2020 were included in our study. Body weight, body length, occipitofrontal head circumference (OFC), and transvertical (vEED) and transfontanellar ear-to-ear (fEED) distances were measured repeatedly. Head eccentricity (ECC) (a measure of disproportional head growth) and head volume indices (HVI) were calculated. Anthropometric data were z-transformed. A total of 367 infants were followed up for assessment of neurodevelopmental outcomes using the Bayley Scales of Infant and Toddler Development (third edition). Associations between cumulative cap time and anthropometric data were examined using unconditional growth models with linear mixed effects. Associations between head growth development and neurodevelopmental outcome were examined by correlating individual regression slopes of anthropometric data with Bayley scores.

**Results:**

Cumulative cap time was negatively associated with z-scores of OFC ($\beta =-1.32\times 10^{-2}$, p<0.005), vEED ($\beta =-6.65\times 10^{-3}$, p<0.005], fEED ($\beta =-1.05\times 10^{-3}$, p>0.05), and HVI ($\beta =-1.59\times 10^{-2}$, p<0.005), while it was positively associated with ECC ($\beta =5.18\times 10^{-3}$, p<0.005). Individual OFC z-score slopes show low correlation with cognition (*R* = 0.07), language (*R* = 0.06), and motor (*R* = 0.01) Bayley scores. Individual vEED z-scores slopes show low correlation with cognition (*R* = −0.10), language (*R* = −0.08), and motor (*R* = −0.07) Bayley scores.

**Conclusion:**

CPAP caps are associated with vertical and horizontal head growth restriction and altered infant head morphology, as indicated by increasing eccentricity. The correlation of the altered growth pattern with neurodevelopmental outcome was negligible. Our findings have clinical implications for the assessment of head growth development during CPAP therapy.

## 1 Introduction

### 1.1 Infant head growth

The occipitofrontal head circumference (OFC) is routinely measured during child development (1). It is a rapid, safe, simple, and economic screening method that reflects both skull volume and brain size (2, 3). Brain growth is the result of glial cell proliferation, myelination, dendrite growth with formation of the dendrite tree, and the establishment of synaptic connections (4). Thus, a physiological increase in head circumference indicates steady development of the brain including sensorimotor, cognitive, and psychological functions (5). Subnormal head growth is associated with impaired neurological development and cognitive abilities (5–8). Especially in the early period after hospital discharge, head growth is a decisive factor for neurodevelopmental outcomes (6, 9). Growth deviations are commonly detected by comparing OFC measurements at various developmental stages with country- and gender-specific reference charts (10–12). However, because OFC does not capture vertical head growth, ear-to-ear distances (EEDs) have been suggested as complementary measurements (13). Recently, growth charts have been established for transfontanellar EED (fEED) and transvertical EED (vEED) distances and also the head volume index (HVI) (14).

Due to immature lung function and respiratory insufficiency, premature infants frequently require mechanical respiratory support, which is frequently applied as non-invasive continuous positive airway pressure (CPAP) via nasal prongs or masks (15–17). Secure CPAP fixation in non-sedated, mobile infants is commonly ensured by snug-fitting CPAP caps (17).

As preterm infants are particularily at risk of developing head deformities due to their malleable bone structure (18), a relationship between these caps and OFC growth below corresponding trajectories, along with simultaneous vertical distortion of the head, has been suggested (19).

On the other hand, OFC growth has been suggested as a benchmarking parameter for preterm infant care because of its postulated relation ship with nutritional therapy and brain development (20–23). However, its OFC growth is influenced by CPAP caps, its association with may be limited.

### 1.2 Aims of this study

It remains unclear whether slow OFC growth in CPAP-treated children accurately reflects slowed head growth and impaired brain development. We hypothesized that tight-fitting CPAP caps, which are commonly open at the top, exert circular compression on the infant skull and restrict OFC growth while simultaneously facilitating compensatory vertical head growth. We further hypothesized an association of this putatively altered head growth pattern with neurodevelopmental outcomes.

## 2 Patients and methods

### 2.1 Patients and settings

In a longitudinal observational study conducted from 2009 to 2020, the body weight, body length, OFC, fEED, and vEED of all patients admitted to our neonatal wards were measured repeatedly from birth to hospital discharge. The mode of ventilation and wearing of a CPAP cap were continuously recorded. Data were documented in routine electronic patient records. The number of observations and the time intervals between measurements for each parameter are summarized in Supplementary Figure S1.

Our study was designed in 2017, with subsequent data collected prospectively. In addition, we included retrospective data from electronic patient records dating back to 2009. Overall, 30.12% of the overall observations were collected prospectively, whereas 69.88% were collected retrospectively. For OFC measurements, 71.05% of observations were collected retrospectively, whereas 28.95% were collected prospectively. For vEED measurements, 69.81% of the observations were collected retrospectively, whereas 30.19% were collected prospectively. Apart from a few retrospective observations, fEED measurements were collected after the beginning of the study, with 96.82% collected prospectively. For body weight, 69.74% of observations were collected retrospectively, while 30.26% were collected prospectively. For body length, 68.82% of the observations were collected retrospectively, while 31.18% were collected prospectively. Our study included 4590 infants, of which 2574 were boys and 2015 were girls. The gender of one infant remained undetermined.

### 2.2 Anthropometry

OFC, fEED, and vEED were measured using a tape as previously described (14). HVI was calculated according to previously described methods (14).

To better assess morphological development of the infant head, we derived geometric surrogate indices from OFC and vEED measurements by assuming an ellipsoidal head shape. First, vertex height (VH) over the coronal plane at the height of the infant’s ear was calculated using Euler’s approximation for the circumference of an ellipse:

$$\mathrm{VH}=\frac{\sqrt{8\times {\mathrm{vEED}}^{2}-{\mathrm{OFC}}^{2}}}{2\pi }$$

The numerical eccentricity (ECC), which describes the deviation of the assumed elliptic head shape from a circle in the frontal plane, was then calculated by

$$\mathrm{ECC}=\sqrt{1-\frac{{\mathrm{vEED}}^{2}}{4\pi \times \mathrm{VH}}}$$

### 2.3 Neurodevelopmental outcomes

Infants born after 2013 were assessed for neurodevelopmental outcomes using the Bayley Scales of Infant and Toddler Development (third edition) (24). Selection criteria for follow-up examination included a birth weight below 1500 g or evidence of perinatal asphyxia, which is defined as cardiorespiratory and neurological depression with an Apgar score <7 at 5 min after birth and acute hypoxia with acidaemia with blood pH <7 or base excess >12 mmol/L (25). Intraventricular hemorrhage (IVH) was assessed by routine ultrasound neuroimaging. A total of 367 infants were eligible for follow-up, of which 231 had a birth weight below 1500 g. Among the 3076 infants born after 2013, 688 had a birth weight below 1500 g. The follow-up rate for children with a birth weight below 1500 g was 33.6%, and the overall follow-up rate was 53.4%.

### 2.4 Statistical analysis and data visualization

Anthropometric data and HVI were z-transformed separately for each gender using previously established generalized additive models for location, scale, and shape (GAMLSS), which were based solely on birth anthropometric data and thus are not influenced by postnatal health and care (14). For ECC z-transformation, we used the birth anthropometric data of Arnold et al. (14) to generate GAMLSS models. We applied the method by Cole and Green (26, 27), and the curves were smoothed using penalized beta splines. We then used these models to z-transform our ECC values utilizing the GAMLSS package (28). The respective ECC centiles are shown in Supplementary Figure S2.

We fitted unconditional growth models with linear mixed effects to our data. Z-scores of OFC, vEED, fEED, ECC, and HVI were used as response variables. Cumulative cap time, gestational age, and z-scores of body length and weight were used as predictor variables. Patient identity was treated as a random effect. We introduced random intercepts and slopes for time and cumulative cap time. The method of Nakagawa et al. (29) was used to calculate conditional pseudo-*R*^2^ for multivariate models. Intraclass correlations (ICC) were computed from the mixed effects model. Variance inflation factors below 5 were considered acceptable. *P*-values <0.05 were considered statistically significant (*p-value <0.05; **p-value <0.01; and ***p-value <0.001.)

Head growth development in infants available for follow-up examination was assessed by calculating regression slopes for the z-scores of repetitively measured anthropometric data using linear regression models. Associations between head growth development and neurodevelopmental outcomes were tested using Pearson’s correlation between individual mean anthropometric z-scores and z-score slopes. To account for the IVH incidence in the follow-up cohort, we fitted generalized multivariate regression models with Bayley scale standard values as response variables and IVH grade, gestational age, gender, and OFC z-score slopes as predictor variables.

Statistical analysis and data visualization were performed using the R software environment, version 4.3.3 (30).

## 3 Results

### 3.1 Cohort characteristics

Clinical characteristics are summarized in Table 1. The study population included a total of 4590 infants, of whom 2574 were boys and 2015 were girls. The gender of one infant remained undetermined, and this infant was excluded from further analysis. The median overall cap time was 12 days, with an interquartile range (IQR) of [4 and 28 days]. Clinical characteristics of the subcohort of 376 patients with available neurological follow-up data are presented in Table 2. The population included a total of 376 patients, of whom 192 were boys and 175 were girls. The median gestational age was 209 days, with an IQR of [193 and 225 days]. The median cap time was 13 days, with an IQR of [4 and 28 days]. There was complete overlap between the OFC and fEED values retrospectively extracted from the clinical records and those determined prospectively from 2017 onwards (Supplementary Figures S3–S5). Thus, there was no need for separate analyses.

**Table 1:** Summary of patient characteristics.

Total patients *N* = 4950, girls 2015 (43.9%), boys 2574 (56.1%), gender not assigned 1 (<0.01%)								
Parameter	Number of infants	Number of observations	Minimum	$P_{25}$	Median	Mean	$P_{75}$	Maximum
Gestational age (days)	4590	4590	161	223	244	243	270	298
Birth weight (g)	4586	4586	350	1600	2330	2370	3130	6110
Birth length (cm)	4481	4481	25.0	34.5	39.0	39.5	44.0	59.0
Birth OFC (cm)	4435	4435	16.0	24.5	27.5	27.8	31.0	45.5
Birth vEEF (cm)	3430	3430	9.5	14.5	16.0	16.4	18.0	41.5
Birth fEED (cm)	806	806	11.0	15.0	17.0	17.7	19.0	35.0
Weight (g)	4586	55977	350	1550	2070	2121	2560	6500
Length (cm)	4481	14170	25.0	40.5	44.5	43.9	47.5	64.0
OFC (cm)	4435	16721	16.0	28.0	31.0	30.4	33.0	45.5
vEED (cm)	3430	9676	9.5	16.0	18.0	18.0	19.5	41.5
fEED (cm)	806	2014	11.0	17.0	18.0	18.4	20.0	35.0
Cap time (days)	4950	4590	0	4	13	18	28	107
Length of stay (days)	4590	4590	0	5	16	25	35	244

$P_{25}$, 25th percentile; $P_{75}$, 75th percentile.

**Table 2:** Summary of characteristics of patients followed up for neurodevelopmental outcomes.

Total patients *N* = 367, girls 175 (47.7%), boys 192 (52.3%)						
Parameter	Minimum	$P_{25}$	Median	Mean	$P_{75}$	Maximum
Gestational age (days)	161	193	209	209	225	290
Age at test (days)	142	175	182	184	191	232
Birth weight (g)	420	906	1300	1342	1728	4090
Birth length (cm)	27.0	34.5	38.0	38.0	41.3	54.0
Birth OFC (cm)	19.5	24.6	27.0	27.0	29.5	37.5
Birth vEEF (cm)	12.0	14.0	16.0	15.8	17.0	22.0
Birth fEED (cm)	11.0	15.0	16.0	16.5	18.0	22.0
Cap time (days)	0	4	14	18	28	65
Length of stay (days)	5.3	32	49	53	71	162
APGAR 10’	4	8	8	8.3	9	10
Bayley III scales (standard values)						
Motor	45	92	103	101	109	137
Language	45	84	100	95	111	155
Cognition	55	85	100	93	105	135
Intraventricular hemorrhage						
Grade	0	1	2	3	4	No data
*N* (percentage)	306 (83.3%)	27 (7.4%)	22 (6.0%)	11 (3.0%)	0 (0%)	1 (0.3%)

$P_{25}$, 25th percentile; $P_{75}$, 75th percentile.

### 3.2 CPAP caps are associated with restricted head growth development

**Figure 1:**
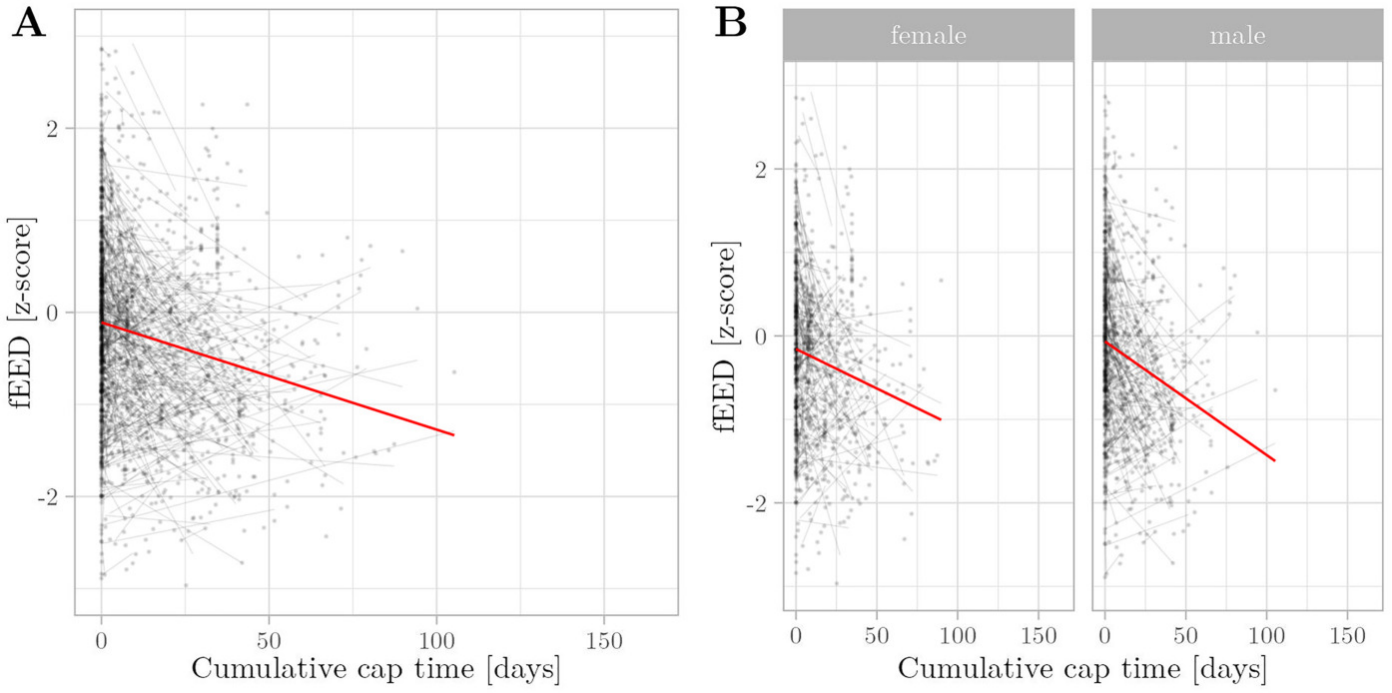
Relationship between cumulative cap time and OFC z-scores was assessed using multivariate regression with linear mixed effects. Semitransparent black dots represent individual measurements, and semitransparent black lines represent individual regression fits. The red line represents the overall model prediction. **(A)** Overall model. **(B)** Separate models for gender.

**Figure 2:**
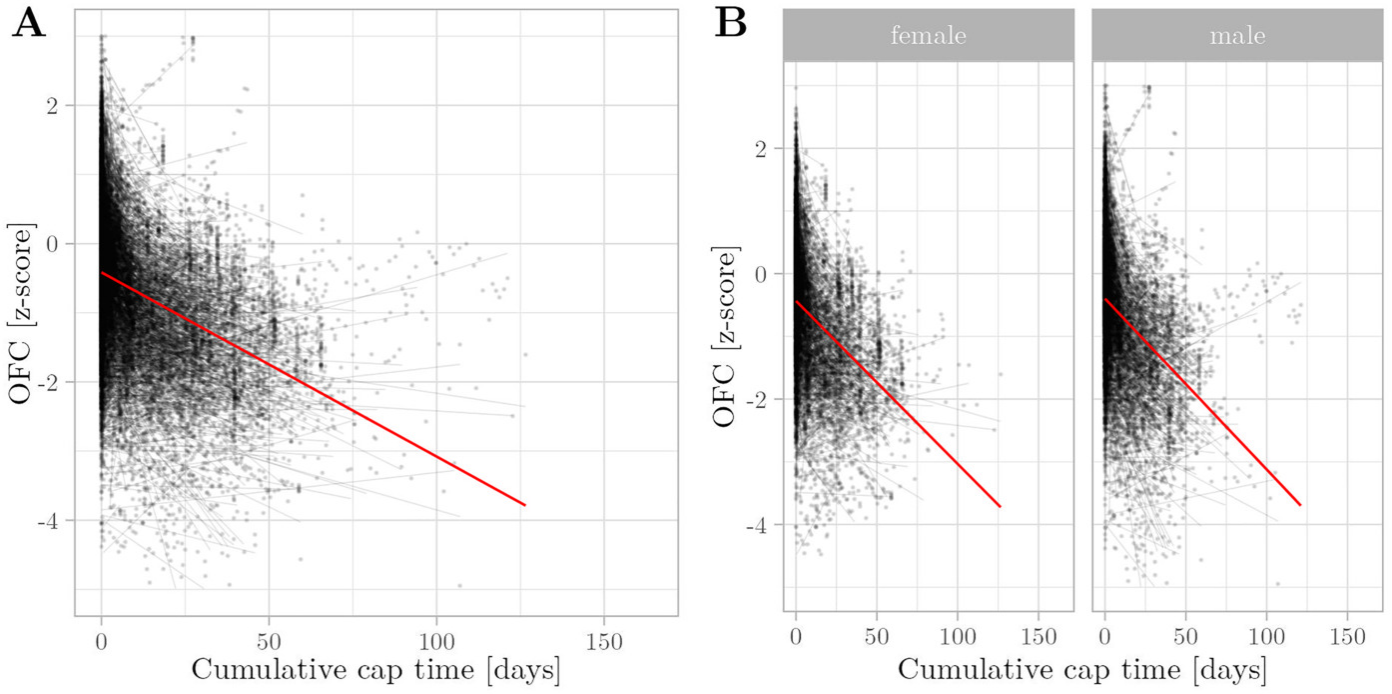
Relationship between cumulative cap time and vEED z-scores was assessed using multivariate regression with linear mixed effects. Semitransparent black dots represent individual measurements, and semitransparent black lines represent individual regression fits. The red line represents the overall model prediction. **(A)** Overall model. **(B)** Separate models for gender.

**Figure 3:**
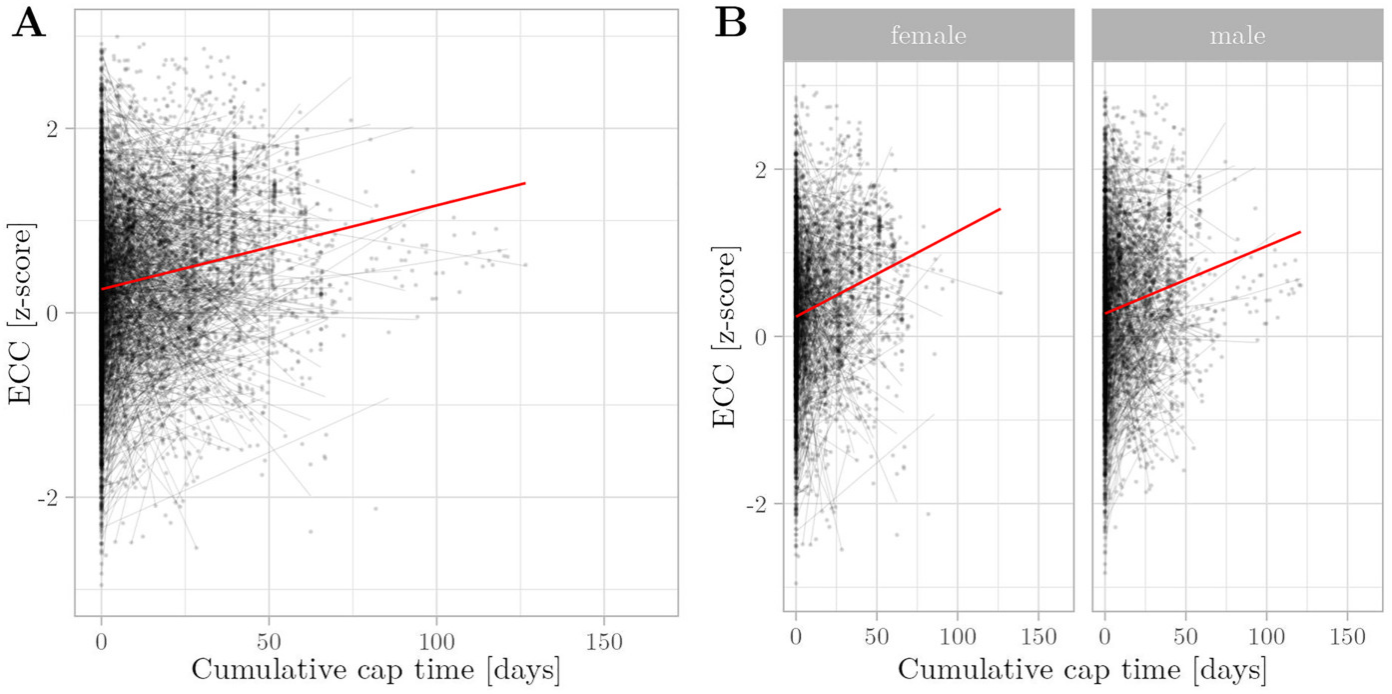
Relationship between cumulative cap time and fEED z-scores was assessed using multivariate regression with linear mixed effects. Semitransparent black dots represent individual measurements, andsemitransparent black lines represent individual regression fits, and the red line represents the overall model prediction. **(A)** Overall model. **(B)** Separate models for gender.

In accordance with our hypothesis, our growth models consistently showed a negative association between cumulative cap time and z-scores of OFC ($\beta =-1.32\times 10^{-2}$, p<0.005) (Figure 1), while gestational age ($\beta =3.59\times 10^{-3}$, p<0.005), body weight ($\beta =6.24\times 10^{-1}$, p<0.005), and body length ($\beta =1.49\times 10^{-1}$, p<0.005) are positively associated. In contrast to our hypothesis, cap time was also negatively associated with vEED ($\beta =-1.05\times 10^{-3}$, p<0.005) (Figure 2), while the association with fEED was not significant ($\beta =-1.05\times 10^{-3}$, p>0.05) (Figure 3). Gestational age was a positive predictor for both vEED ($\beta =6.39\times 10^{-3}$, p<0.05) and fEED ($\beta =4.26\times 10^{-3}$, p<0.05) z-scores. Weight and length development were significant positive predictors for vEED z-scores ($\beta =6.24\times 10^{-1}$, p<0.005 and $\beta =1.49\times 10^{-1}$, p<0.005, respectively). Weight gain was also positively associated with fEED z-scores ($\beta =3.51\times 10^{-1}$, p<0.005), while the association with length was not significant ($\beta =8.10\times 10^{-2}$, p>0.05). Interestingly, the infants’ gender was not significantly associated with head growth development (Table 3). These results indicate CPAP caps restrict vertical and horizontal head growth.

**Table 3:** Multivariate regression analysis of an unconditional growth model with linear mixed effects.

Parameter	β-coefficients				
	z(OFC)	z(vEED)	z(fEED)	z(ECC)	z(HVI)
Cap time	$-1.32\times 10^{-2}$	$-6.65\times 10^{-3}$	$-1.5\times 10^{-3}$	$5.18\times 10^{-3}$	$-1.59\times 10^{-2}$
(days)	$\left(7.60\times 10^{-4}\right)$***	$\left(1.20\times 10^{-3}\right)$***	$\left(2.80\times 10^{-3}\right)$	$\left(1.33\times 10^{-3}\right)$***	$\left(1.08\times 10^{-3}\right)$***
Gestational age	$3.59\times 10^{-3}$	$6.39\times 10^{-3}$	$-4.26\times 10^{-3}$	$-2.56\times 10^{-3}$	$4.57\times 10^{-3}$
(days)	$\left(3.69\times 10^{-4}\right)$***	$\left(5.92\times 10^{-4}\right)$***	$\left(1.74\times 10^{-3}\right)$*	$\left(5.89\times 10^{-4}\right)$***	$\left(4.68\times 10^{-4}\right)$***
Male	$3.58\times 10^{-4}$	$9.59\times 10^{-3}$	$9.83\times 10^{-3}$	$-4.11\times 10^{-2}$	$9.83\times 10^{-3}$
(gender)	$\left(2.23\times 10^{-2}\right)$	$\left(3.72\times 10^{-2}\right)$	$\left(1.06\times 10^{-2}\right)$	$\left(3.66\times 10^{-2}\right)$	$\left(2.92\times 10^{-2}\right)$
Weight	$6.24\times 10^{-1}$	$3.97\times 10^{-1}$	$3.51\times 10^{-1}$	$-7.65\times 10^{-2}$	$6.37\times 10^{-1}$
(z-score)	$\left(1.29\times 10^{-2}\right)$***	$\left(2.26\times 10^{-2}\right)$***	$\left(5.02\times 10^{-2}\right)$***	$\left(2.55\times 10^{-2}\right)$**	$\left(1.98\times 10^{-2}\right)$***
Length	$1.49\times 10^{-1}$	$1.41\times 10^{-1}$	$8.10\times 10^{-2}$	$7.96\times 10^{-2}$	$-1.52\times 10^{-1}$
(z-score)	$\left(1.26\times 10^{-2}\right)$***	$\left(2.19\times 10^{-2}\right)$***	$\left(4.75\times 10^{-2}\right)$***	$\left(2.46\times 10^{-2}\right)$***	$\left(1.91\times 10^{-2}\right)$***
$R^{2}$	0.86	0.84	0.93	0.58	0.85
ICC	0.67	0.75	0.91	0.56	0.64
VIF	1.11	1.10	1.08	1.16	1.13

Z-scores of OFC, vEED, fEED, ECC, and HVI were treated as response variables. Cap time, gestational age, gender, and z-scores of weight and length were treated as response variables. β-coefficients are shown followed by standard errors in parentheses. Patient identity was treated as a random effect. We introduced random slopes and intercepts for cap time. Gestational age, gender, and weight and length z-scores were treated as fixed effects. Conditional ${\text{R}}^{2}$ was calculated using the method by Nakagawa. Intraclass correlations (ICC) were computed from the mixed effects mode. Variance inflation factors (VIF) below 5 were accepted.

*p<0.05, **p<0.01, ***p<0.001.

### 3.3 Cap time is associated with altered head morphology

**Figure 4:**
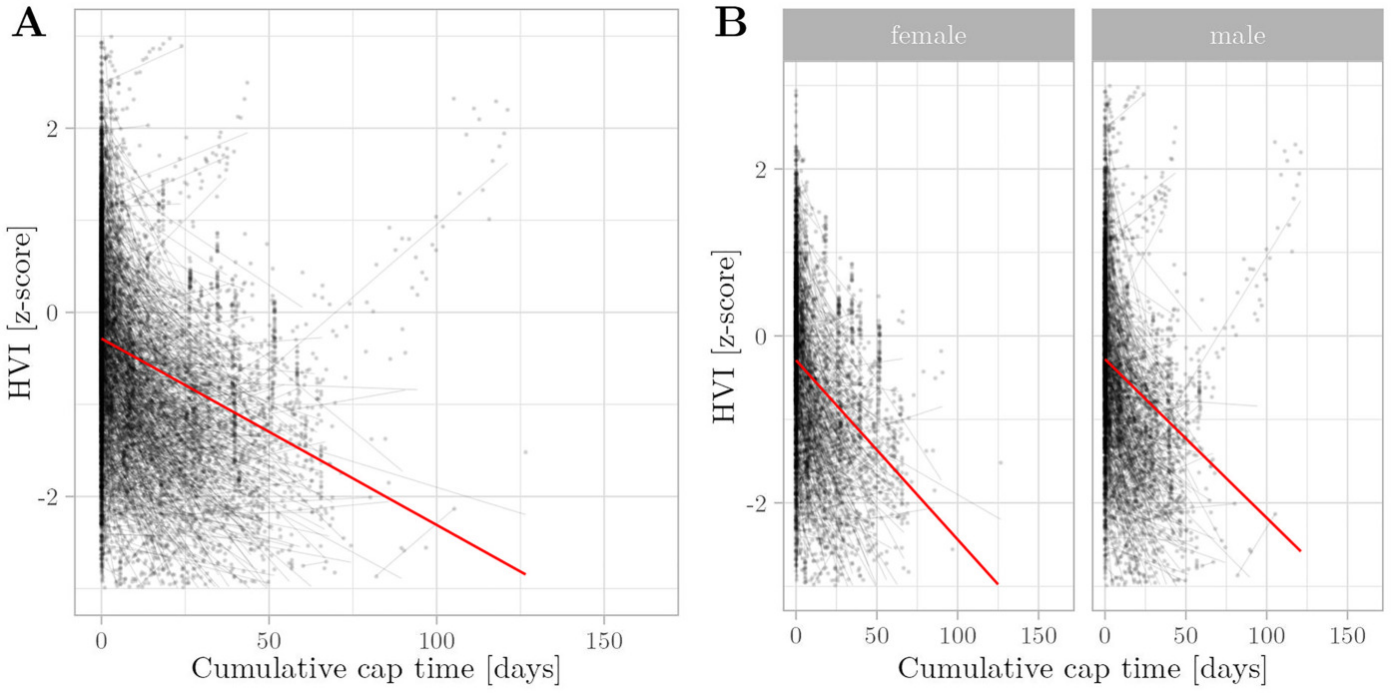
Relationship between cumulative cap time and eccentricity (ECC) z-scores was assessed using multivariate regression with linear mixed effects. Semitransparent black dots represent individual measurements, and semitransparent black lines represent individual regression fits. The red line represents the overall model prediction. **(A)** Overall model. **(B)** Separate models for gender.

Cumulative cap time positively predicted the infant head ECC z-scores ($\beta =5.18\times 10^{3}$, p<0.005). This means that, vEED deviated to a lesser extent from the birth centiles than OFC. Gestational age was thus negatively associated with ECC z-scores ($\beta =-2.56\times 10^{-3}$, p<0.005) (Figure 4). Interestingly, weight and length development had opposing influences on ECC, with weight being a negative predictor ($\beta =-7.65\times 10^{-2}$, p<0.01) and length being a positive predictor ($\beta =7.96\times 10^{-2}$, p<0.01) of ECC z-scores. Infant gender had no significant association with head ECC z-scores ($\beta =-4.11\times 10^{-2}$, p>0.05). Interestingly, cumulative intubation time was significantly associated with stagnant OFC growth, whereas its associations with EED or ECC z-scores were statistically not significant (Supplementary Table S1). These results indicate that CPAP caps are associated with an altered growth pattern, rendering the infant’s head more eccentric.

### 3.4 Altered head morphology does not compensate for head growth restriction

**Figure 5:**
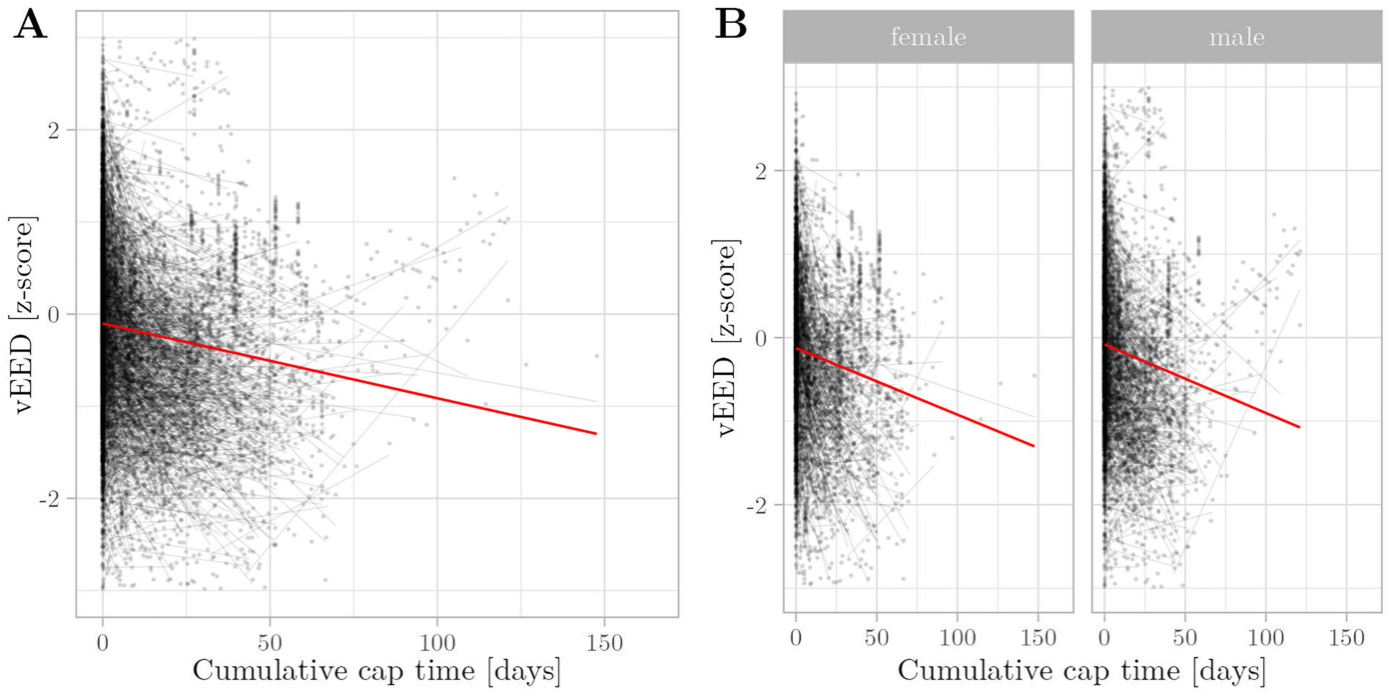
Relationship between cumulative cap time and HVI Z-scores was assessed using multivariate regression with linear mixed effects. Semitransparent black dots represent individual measurements, and semitransparent black lines represent individual regression fits. The red line represents the overall model prediction. **(A)** Overall model. **(B)** Separate models for gender.

Cumulative cap time was negatively associated with HVI z-scores ($\beta =-1.59\times 10^{-2}$, p<0.005) (Figure 5). Gestational age ($\beta =4.57\times 10^{-3}$, p<0.005) and weight development ($\beta =6.37\times 10^{-1}$, p<0.005) were positively associated. Surprisingly, length development reversed this pattern and showed a negative association ($\beta =-1.52\times 10^{-1}$, p<0.005). Gender was not a significant predictor of HVI z-scores ($\beta =9.83\times 10^{-3}$, p>0.05). These results indicate that the infants’ head volume is significantly lower with longer cap time duration and that the eccentric growth pattern associated with CPAP caps does not fully compensate for the restriction in head growth.

### 3.5 Altered head growth is not associated with neurodevelopmental outcomes

Correlations between cognitive abilities and both the mean z-scores and the slopes of both OFC and vEED were minimal and statistically and clinically insignificant (Table 4). Consistently, multivariate generalized models showed significant associations between neurodevelopmental outcomes with IVH grade, gestational age, and gender, while OFC z-score slopes were not significantly associated (Table 5).

**Table 4:** Correlation matrix of cephalometric development and Bayley III scales.

Parameter	Bayley scales of infant and toddler development III (standard values)						
	Cognition	Language overall	Language expressive	Language receptive	Motor overall	Motor gross	Motor fine
Mean OFC (z-score)	0.13	0.08	−0.04	−0.02	0.14	−0.06	−0.03
OFC slope (z-score/day)	0.07	0.06	0.05	0.03	0.01	−0.01	−0.02
Mean EED (z-score)	0.16	0.09	−0.06	−0.03	0.10	−0.16	−0.12
vEED (z-score/day)	−0.10	−0.08	−0.04	−0.10	−0.07	−0.07	−0.05

Pearson’s R correlation coefficients between Bayley Scales of Infant and Toddler Development III and cephalometric development (mean z-scores and individual regression slopes of OFC and vEED).

**Table 5:** Generalized multivariate regression analysis.

Parameter	β-coefficients		
	Cognition	Language	Motor
IVH 1	−4.64 (3.38)	−3.48 (4.25)	−2.97 (3.07)
IVH 2	−7.93 (3.78)*	−4.75 (4.75)	−9.37 (3.43)**
IVH 3	−12.35 (5.12)*	−12.01 (6.44)*	−16.80 (4.65)***
Gestational age (days)	0.12 (0.04)**	0.12 (0.06)*	0.07 (0.04)
Male (gender)	−3.09 (1.76)	−5.96 (2.22)**	−3.16 (1.60)*
OFC slope (z-score/day)	74.89 (39.48)	66.5 (49.62)	12.55 (35.86)
$R^{2}$	0.06	0.04	0.07

β-coefficients are shown followed by standard errors in parentheses.

Bayley III scale standard values were treated as response variables. IVH grades, gender, gestational age, and OFC z-score slopes were treated as predictor variables.

*p<0.05, **p<0.01, ***p<0.001.

## 4 Discussion

### 4.1 CPAP caps are associated with an altered head growth pattern

We conducted an observational study to estimate the effect of CPAP caps on head growth development in a large cohort of newborn infants. Head development was compared to normative data that was only based on birth centiles, z-transformed using GAMLSS models, and thus unaffected by postnatal influences. To assess the infant head morphology and avoid covariance between HC and vEED or fEED, we calculated head eccentricities. Our models fit the data very well, with ${\text{R}}^{2}$-values ranging from 0.58 to 0.93.

Low gestational age is associated with impaired head growth. However, low gestational age is also associated with impaired lung function, which in turn requires longer CPAP therapy duration. To control for this confounding effect, we included gestational age and growth and length development as predictor variables in our regression model. To account for multicollinearity, we calculated variance inflation factors (VIF) and excluded predictors if their values exceeded 5.

Our results indicate that CPAP cap use is associated with restricted horizontal and vertical head growth. As cap time and gestational age are both scaled in days, our model allows for a direct comparison of their effect sizes. Interestingly, the negative effect of cap time on OFC z-scores exceeded the positive effect of gestational age by a factor of 3.68. This ratio was less pronounced for vEED, for which the negative effect of cap time and the positive effect of gestational age are approximately equal. The negative effect of cap time on HVI exceeded the positive effect of gestational age by a factor of 3.47. This highlights the relevance of CPAP caps for the evaluation of head growth development in a clinical setting. However, the high ICC values indicate a pronounced effect of infant identity on its anthropometric development.

In our analysis, vEED showed consistent associations with our predictor variables and seems to be a more reliable estimator of vertical head growth than fEED. This appeared plausible, as fEED is measured across the great fontanelle, which is more dynamic in size and consistency. This is also reflected by a high ICC of 0.91 for fEED, compared to an ICC for vEED of only 0.75.

Our model reveals a positive association between CPAP caps use and ECC. However, infant head volume is negatively affected by cap duration. This indicates that eccentric head growth does not fully compensate for OFC growth restriction in terms of head volume. As a dimensionless metric, ECC seems to be a stable tool for the characterization of infant skull morphology. Accordingly, the ICC was the lowest for ECC, which indicates a minimal individual effect. In a clinical setting, it may be too laborious to calculate ECC values manually. A cell phone app may be a remedy to this.

Previous studies using cephalometric stereophotogrammetry have not supported an association between the duration of respiratory support and restrictions in OFC and head volume. Moreover, thse studies report a high prevalence of dolichocephaly among preterm infants (31, 32). Dolichocephaly is determined from the cranial index, which detects occipital elongation and skull narrowing and does not necessarily measure cranial height above the transversal plane. Moreover, these studies did not apply transformation based on normative data and included relatively small numbers of patients.

OFC growth is commonly considered a quality criterion for the care of preterm infants, especially regarding adequate nutrition. Our data reveal that OFC development is strongly influenced by CPAP cap time, suggesting that it may be an inadequate surrogate parameter for assessing nutritional success. To compensate for the distorting effect of CPAP caps on the assessment of patient care practices, we suggest adjusting OFC z-scores to the cumulative cap duration (CD), which may be derived from our model:

$$z(\mathrm{OFC}{)}_{\mathrm{adjusted}}=z(\mathrm{OFC}{)}_{\mathrm{measured}}-(\;-0.0132\times \mathrm{CD}\;[\mathrm{days}])$$

Further research and clinical experience are needed to assess whether this correction allows more accurate benchmarking of preterm infant care. As our model shows a high individual effect on OFC development (ICC = 0.67), this correction must be used carefully. Alternatively, ECC or HVI may represent more accurate benchmarking parameters, as they combine horizontal and vertical head growth into a single measure.

HVI seems suitable for following head growth development in preterm infant care, as it is easy to calculate and reflects both infant head morphology and absolute infant head volume. Our data showed a negative association with CPAP cap use, which suggests that prolonged CPAP cap use is associated with a smaller head volume. Furthermore, the effect of CPAP cap time on HVI and ECC was concordant, which suggests that HVI also detects alterations in infant head morphology. Although ECC is more accurate in describing infant head morphology, it is a dimensionless index that does not reflect head size and thus does not indicate micro- or macrocephaly.

Recently, automatic head shape analysis for the detection of cranial deformities and head growth disturbances has been developed (33–35). These innovations are of high clinical interest and may be more complex but also more accurate tools for the assessment of infant head growth development.

### 4.2 Altered growth patterns are not correlated with neurodevelopmental outcomes

The effect of fetal head growth on neurodevelopmental outcomes has been previously investigated in several observational studies (6–9, 36, 37), which were conducted across various decades (ranging from 1982 to 2014) and various geographic locations. However, none of these studies considered the influence of CPAP therapy, which was first applied in 1971 (38) but came into broader clinical use during the last 20 years. Even later, CPAP caps were introduced as a more comfortable and secure method for affixing prongs or masks to the infant’s nose. OFC growth during the first year of life has been shown to predict early child behavioral traits in healthy populations. Postnatal OFC growth has been found to negatively predict temperamental surgency/extroversion and effortful control and positively predict gross motor skills in healthy boys at 24 months of age. No significant effect of OFC growth was found in girls (36). Postnatal head growth may have a more important role in determining cognitive outcome than intrauterine head growth (6, 9). Poor growth in early life may not be compensated for during infancy and childhood (39). Moreover, small OFC at birth and poor postnatal OFC growth are associated synergistically with a higher risk of neurodevelopmental impairment at 2 years of corrected age in preterm infants, especially affecting fine motor and coordination domain (37).

To assess the effect of the observed altered growth pattern on neurodevelopmental outcomes, we calculated individual regression slopes of head measurement z-scores and correlated them with Bayley III scores for a subset of patients. As cumulative cap time is also an independent indicator of individual disease severity, which presumably affects the neurodevelopmental outcome negatively, we chose this approach. We found negligible correlations of z-score slopes and mean z-scores with Bayley III scores.

A major limitation of this study is that the selection criteria for follow-up inherently include risk factors for clinical complications and impaired neurodevelopment. Moreover, infants with high disease severity may not be represented due to death before follow-up or ineligibility caused by medical or custodial impediments. However, within that group at risk for suboptimal neurodevelopment, it is possible to characterize associations between head growth development, clinical complications, and neurodevelopmental outcomes. To take our selection bias into account, we included ultrasound neuroimaging data in our analysis and found signifiacnt associations between IVH grade, gestational age, and gender, while OFC development was not signifincantly associated. This underpins that the observed altered growth pattern, caused by CPAP caps, was not associated with poorer developmental outcomes. This finding may provide reassurance for clinicians managing cases with slowed head growth during CPAP therapy.

### 4.3 Limitations

It remains unclear whether the observed altered growth pattern and eccentric skull morphology represent a stable condition or whether there is catch-up growth and head reformation after the cessation of CPAP treatment. This question must be addressed in future follow-up studies. Moreover, neurodevelopment was assessed at an early stage in childhood development. Neurodevelopmental impairments manifesting at later stages caused by slowed head growth in infancy cannot be excluded, although we consider it unlikely.

A major limitation is the observational design of our study, which does not allow for causal inferences, although we consider our conclusions to be mechanistically plausible. We cannot exclude confounding factors likely associated with the need for extended CPAP therapy such as parental socioeconomic status or individual disease severity. To mitigate these individual effects, we included patient identity as a random effect and introduced random slopes and intercepts for cumulative cap time. We quantitatively estimated the individual effects on the observed variance by calculating intraclass correlation coefficients (Table 3).

Our study included both prospective and retrospective data. We found no qualitative difference between the prospective and retrospective cohorts (Supplementary Figures S3–S5).

## 5 Conclusion

CPAP cap use was associated with reduced head growth in early infancy, restricting horizontal head growth and overall head volume, and, to a lesser extent, vertical head growth. Thereby, they rendered infants’ heads more eccentric. This altered growth pattern was not correlated with neurodevelopmental outcome in early infancy. These findings may have clinical implications for the evaluation of stagnant head growth during CPAP therapy in preterm infant care and early infancy.

## Data Availability

The datasets presented in this article are not readily available because raw data sharing was not part of the IRB application and thus not approved. Requests to access the datasets should be directed to Professor Ulrich Thome, ulrich.thome@medizin.uni-leipzig.de.
