# Supplementary material for: CPAP caps are associated with restricted head growth and altered skull morphology in newborn infants
Source: Front Pediatr. 2025 May 19;13:1514853. doi: 10.3389/fped.2025.1514853 (PMC12128420; doi:10.3389/fped.2025.1514853)
Supplement: Supplementary file 1 [file Datasheet1.pdf]

Supplementary Material

Histograms for the number of observations per patient and the time intervals between two measurements of anthropometric data are shown in figure S1. ECC centiles are shown in figure S2. We compared the retrospective cohort (from 2009 to 2016) with the prospective cohort (from 2017-2020). A histogram and density plot of birth weight and gestational age is shown in figure S3. Scatterplots of anthropometric data are shown in figure S4. To assess the quality and measurement error of the recorded data, we calculated individual consecutive z-score differences and plotted them as violin plots in figure S5.

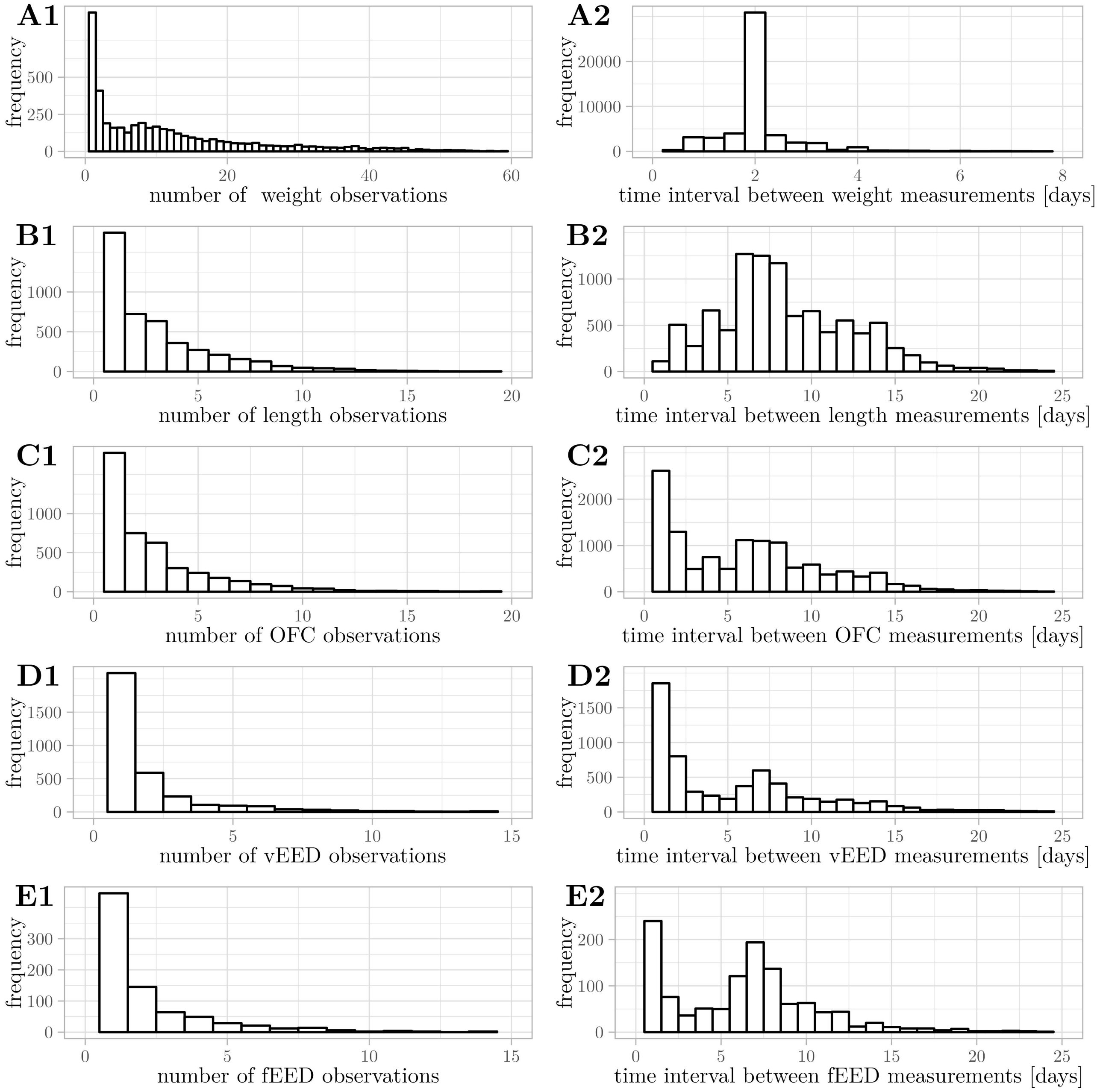

Figure S1: Histograms for the number of observations per patient and the time intervals between two measurements for weight (A1 and A2), length (B1 and B2), OFC (C1 and C2), vEED (D1 and D2) and fEED (E1 and E2).

Supplementary table: Multivariate regression analysis of an unconditional growth model with linear mixed effects. Z-scores of OFC, vEED, fEED, ECC and HVI were treated as response variables. Intubation time, gestational age, gender and z-scores of weight and length were treated as response variables. β-coefficients are shown followed by standard error in brackets. Patient identity was treated as a random effect. We introduced random slopes and intercepts for intubation time. Gestational age, gender and weight and length z-scores were treated as fixed effects. * p <0.05. ** p <0.01. *** p <0.001.

	β-coefficients				
parameter	z(OFC)	z(vEED)	z(fEED)	z(ECC)	z(HVI)
intubation time [days]	-3.05×10^{-2} (5.67×10^{-3}) ***	-1.98×10^{-2} (1.50×10^{-2})	-4.13×10^{-2} (3.82×10^{-2})	2.78×10^{-2} (1.64×10^{-2})	-4.08×10^{-2} (1.18×10^{-2}) ***
gestational age [days]	1.61×10^{-2} (4.13×10^{-3}) ***	4.81×10^{-3} (1.39×10^{-2})	-3.86×10^{-1} (1.82×10^{-1}) *	1.45×10^{-3} (1.46×10^{-2})	3.57×10^{-3} (1.06×10^{-2})
male [gender]	1.88×10^{-2} (2.96×10^{-2})	-1.79×10^{-3} (4.54×10^{-2})	-1.00×10^{-2} (1.10×10^{-1})	-8.06×10^{-3} (4.79×10^{-2})	-3.44×10^{-2} (3.49×10^{-2})
weight [z − score]	5.96×10^{-1} (2.17×10^{-2}) ***	3.88×10^{-1} (3.40×10^{-2}) ***	3.99×10^{-1} (7.05×10^{-2}) ***	-6.81×10^{-2} (3.61×10^{-2})	5.93×10^{-1} (2.62×10^{-2}) ***
length [z − score]	9.52×10^{-2} (2.17×10^{-2}) ***	1.56×10^{-1} (3.30×10^{-2}) ***	1.51×10^{-1} (6.79×10^{-2}) *	1.19×10^{-1} (3.50×10^{-2}) ***	1.40×10^{-1} (2.54×10^{-2}) ***

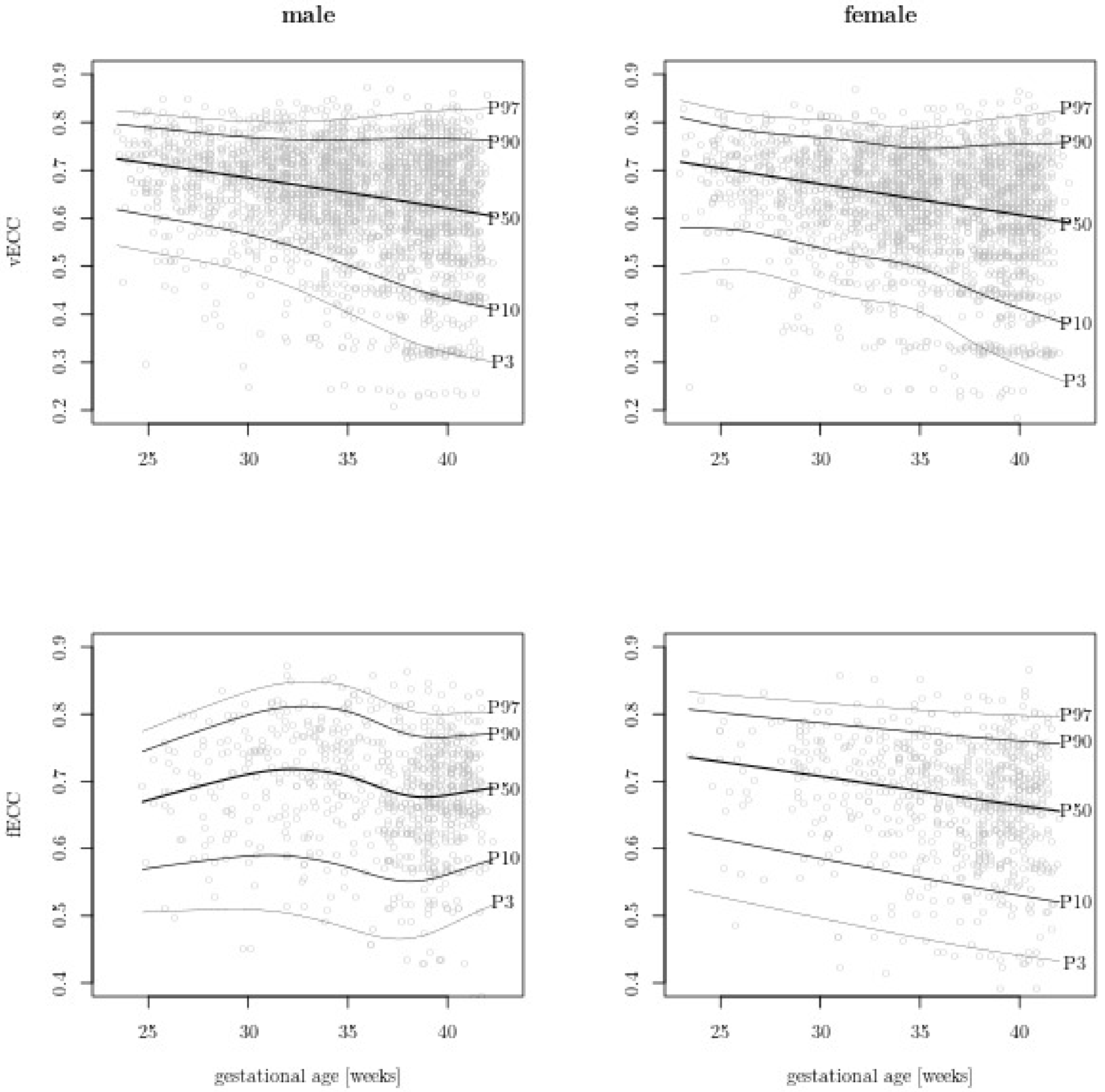

Figure S2: Birth centiles of vertical and fontanellar numeric eccentricity (ECC) for male and female infants. Centiles were calculated from birth anthropometric data by GAMLSS models using the method of Cole and Green and smoothing with penalized beta splines

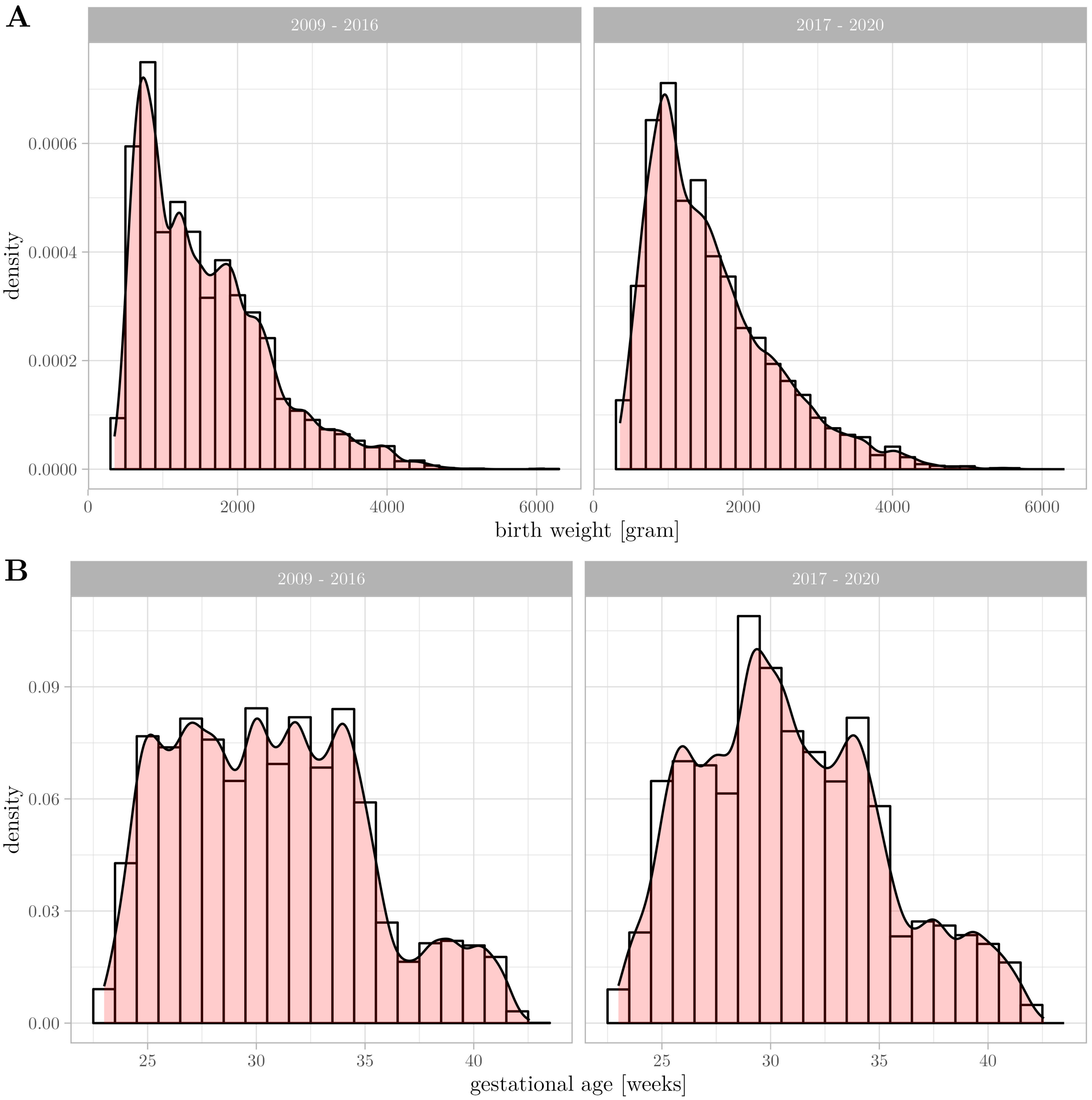

Figure S3: Histogram and density plot for birth weight (A) and gestational age (B) of the prospective (2017-2020) and the retrospective (2009-2016) cohort.

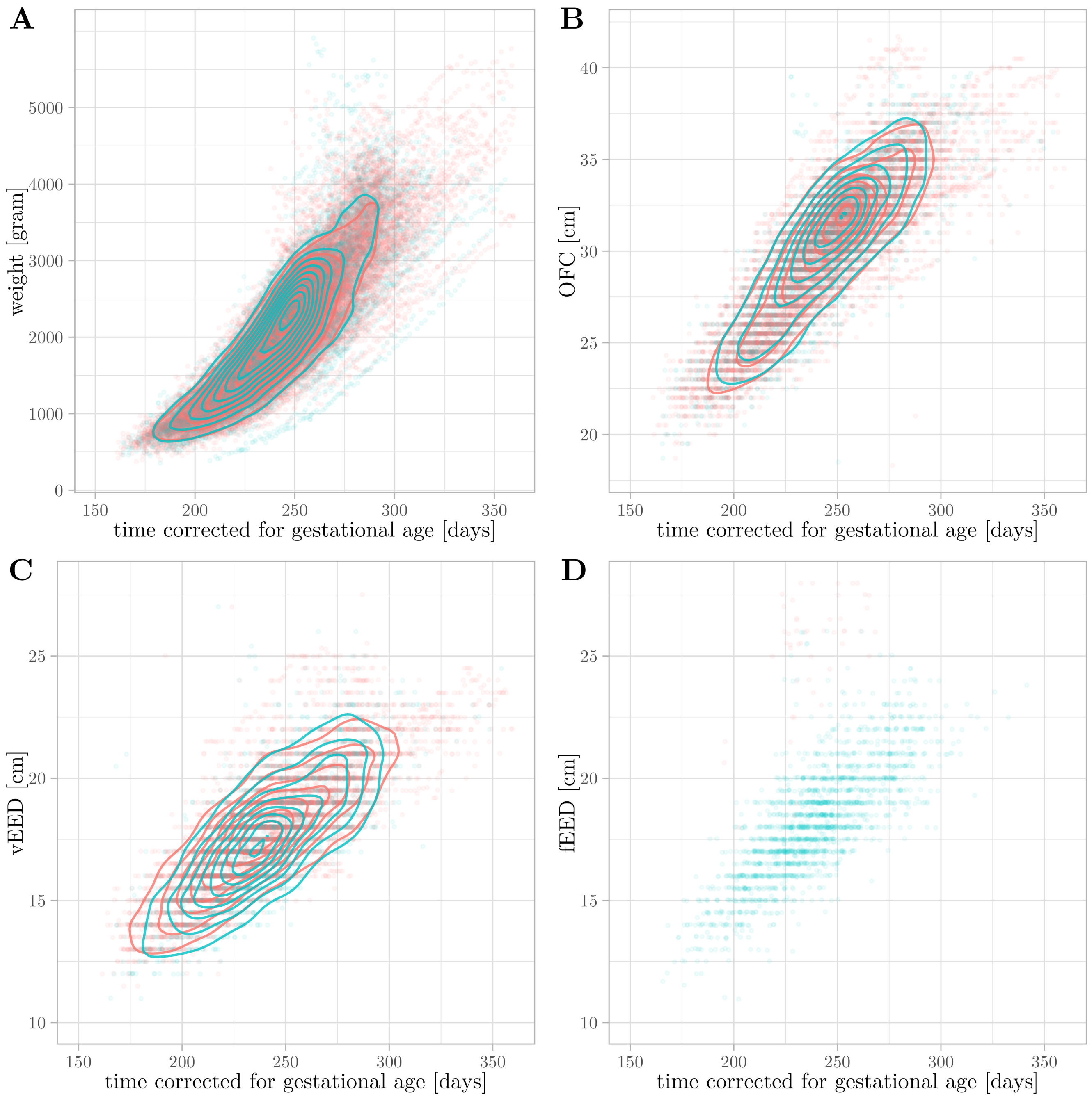

Figure S4: Scatterplot and density estimate of weight (A), OFC (B), vEED (C) and fEED (D) against time corrected for gestational age. The red colour indicates the retrospective cohort (2009-2016), the blue colour indicates the prospective cohort (2017-2020). As fEED was measured almost exclusively in the prospective cohorts, the density estimate is missing in D.

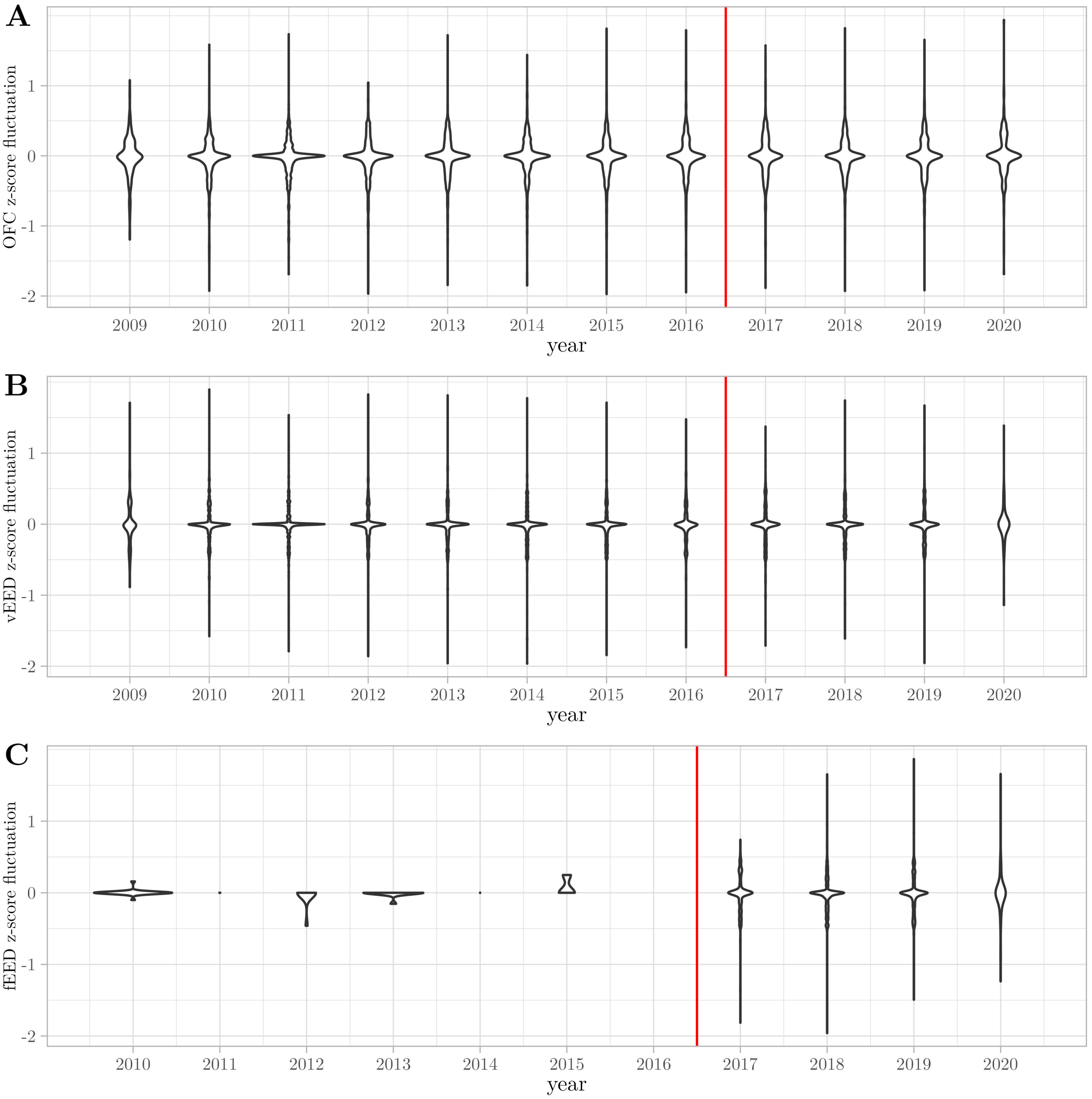

Figure S5: Violin plot of consecutive individual z-score differences (z-score fluctuations) for OFC (A), vEED (B) and fEED (C) for every year of the study. The red line indicates the distinction between the retrospective (2009-2016) and the prospective (2017-2020) cohort.
